# Trans 10, cis 12-conjugated linoleic acid reduced reproductive ability by disrupting the estrus cycle in female mice

**DOI:** 10.1590/1984-3143-AR2024-0010

**Published:** 2024-05-13

**Authors:** Shuai Yu, Baozhu Wang, Yu Rao, Mei Liu, Luwen Liang, Kemian Gou

**Affiliations:** 1 Jiangsu Co-innovation Center for Prevention and Control of Important Animal Infectious Diseases and Zoonoses, Institute of Comparative Medicine, College of Veterinary Medicine, Yangzhou University, Yangzhou, China

**Keywords:** trans 10, cis 12-conjugated linoleic acid (t10c12-CLA), estrous cycle, fertility, progesterone

## Abstract

As a positional and geometrical isomer of linoleic acid, trans 10, cis 12 conjugated linoleic acid (t10c12-CLA) reduces white fat by reducing food intake, modulating lipid metabolism, and stimulating energy expenditure. However, the t10c12-CLA products are mostly mixtures, making it difficult to obtain accurate results. Studies are needed to investigate the effects of pure t10c12-CLA on animals and humans. In this study, we used the biallelic transgenic (tg) mice, which could produce t10c12-CLA itself, to investigate the effects of pure t10c12-CLA on female reproductive ability. The results showed that the body and relative ovary weights had no significant difference between tg and wild-type (wt) littermates at ages 3 or 10 weeks. While the fecundity test found that tg mice had a significantly longer first litter time (32.0 ± 4.70 days vs. 21.3 ± 2.31 days, *P*<0.05), and a significantly lower number of litters (4.75 ± 2.75 vs. 6.67 ± 0.57, *P*<0.05) when compared with wt mice during continuous mating within seven months. Hormone profiles showed that serum estradiol levels did not change in tg mice; however, significantly (*P*<0.05) decreased progesterone and increased prostaglandin E2 levels were observed in tg mice compared with those of wt mice. Hematoxylin-eosin staining showed no pathological characteristics in tg ovaries, except for the increased atresia follicles (*P*<0.05). Moreover, the tg mice had a significantly more extended diestrus period than the wt mice (48.4 ± 6.38% vs. 39.6 ± 3.81%, *P*<0.05). In summary, t10c12-CLA could affect serum progesterone and prostaglandin E2 levels, lead to a disordered estrus cycle, and impact the reproductive performance of female mice. This study provided theoretical and biosafety recommendations for applying t10c12-CLA in female mammals.

## Introduction

The impact of female reproductive health on pregnancy rates and offspring has garnered significant attention. Female reproductive factors, such as ovarian development, oocyte maturation, estrous cycle, and embryo implantation, were influenced primarily by sex hormones, including progesterone, prostaglandin, and estrogen (Innocenti et al., 2017; Bui et al., 2022). Sex hormones play crucial roles in the complex and interconnected processes of pregnancy and childbirth (Zamberlam et al., 2019). Fat could affect the synthesis of cholesterol, which is the precursor of sex hormones (Fox et al., 2009). Moreover, fat metabolism significantly participates in female prenatal and postnatal health by modulating the synthesis and secretion of sex hormones and the physiology of the placenta (Ramakrishnan et al., 2012; Fontana and Della Torre, 2016; Saben et al., 2016; Jazwiec and Sloboda, 2019). Hence, the metabolic processes related to fat played a critical role in the reproductive capacity of females.

Trans 10, cis 12-conjugated linoleic acid (t10c12-CLA), a positional and geometrical isomer of linoleic acid (LA), had received widespread attention as a potential health product. Currently, t10c12-CLA has been shown to play an active role in anti-obesity, anti-cancer, anti-oxidative stress, and immune enhancement due to its involvement in fat metabolism (den Hartigh, 2019; Badawy et al., 2023). CLA could also affect female mice’s reproductive performance by reducing FSH and LH levels in the blood (Badawy et al., 2023). While gastric administration of CLA mixtures during the pregestational and gestational periods did not affect the offspring’s ovarian follicle endowment and mobilization, nor did it affect oocyte lipid accumulation, demonstrating that this supplement was without detrimental effects on female reproductive healthy mice (Freitas et al., 2023). Supplementation with CLA mixtures in cows increased circulating progesterone, insulin-like growth factor-1, and leptin, improved fertility, and reduced the period from calving to conception (Csillik et al., 2017). It was controversial that the female reproductive ability was enhanced by CLA supplementation by these results. This is because the usage of CLA was a mixture of CLA: t10c12-CLA and cis 9, trans 11-CLA (c9t11-CLA), which caused doubts about the credibility of the results (den Hartigh, 2019).

Since CLA products were mostly mixtures and pure t10c12-CLA was easily oxidized in the external environment, it took time to obtain accurate results successfully, even conflicting results. Propionibacterium acnes isomerase (Pai) could successfully convert linoleic acid (LA) into t10c12-CLA in recombinant lactic acid bacteria and murine 3T3 cells (Li et al., 2015; Wang et al., 2021). Then, a novel transgenic mouse was produced in our lab by introducing the Pai-expressing cassette into the Rosa26 locus of C57BL/6 mice using CRISPR-Cas9 technology (Rao et al., 2023a, b). The transgenic (tg) mouse could synthesize t10c12-CLA by itself. The male mouse could produce t10c12-CLA in various tissues throughout the body while increasing heat production and decreasing fat accumulation (Rao et al., 2023a). This is consistent with the anti-obesity results from oral t10c12-CLA administration, suggesting that the mouse can be used to investigate the impact of t10c12-CLA in mice. T10c12-CLA could induce changes in levels of many hormones in female tg mice, predicting that t10c12-CLA could affect individual development, predominantly female reproductive performance (Rao et al., 2023b). The female mouse also provided an excellent experimental model to explore the regulation of t10c12-CLA on female reproduction.

To assess the impact of pure t10c12-CLA on biometric parameters, ovarian morphology and physiology, as well as sex hormone levels, tg mice were utilized to monitor alterations in female reproductive function resulting from prolonged existence to t10c12-CLA. This study provided theoretical and biosafety recommendations for applying t10c12-CLA in female mammals.

## Methods

### Mice

Animal procedures were approved by the Animal Care and Use Committee of Yangzhou University by the Guide for the Care and Use of Laboratory Animals of the National Institutes of Health. The animal study protocol was approved by the Ethics Committee of Yangzhou University (Protocol Code NSFC2020-SYXY-20 and dated 25 March 2020). The C57BL/6J mice were obtained from the Laboratory Animal Centre, Yangzhou University, China, and used as wild-type (wt) mice. The tg mice (C57BL/6J background) were produced by our lab (Rao et al., 2023a, b). Each mouse per cage used in this study was maintained in an environment with a controlled temperature of 22 ± 2 °C, with artificial light cycles (12 h:12 h, lights on at 07:00), and was fed ad libitum with a standard diet containing 10% kcal% fat. The wt and tg female mice used in this study were weighed at 3 weeks and 10 weeks postnatally.

### Assessment of fertility

Each female mouse at the age of 8 weeks postnatally was randomly mated with a wt fertile male at the age of 8 weeks and caged together for over seven months. The male mice were randomly divided into each cage to mate with an 8-week-old female, and marked as Male-wt × Female-wt (wt × wt, the sex ratio: 1:1, n=4) or Male-wt ×Female-tg (wt × tg, the sex ratio: 1:1, n=4). During the period, we accurately and constantly recorded the following parameters: number of litters, litter size, and the sexual ratio of offspring per litter. We also recorded the length of time from first mating to parturition and named it “latency (in days) to the first litter”. The length of time from the other mating to parturition was named “latency (in days) to the other litters” (Nicolakis et al., 2020). The assessment of the fertility experiments was repeated three times and had consistent results.

### Hormones measurement

The blood from the orbital sinus vein was used to measure the serum levels of estradiol, progesterone, and prostaglandin E2 using the ELISA detective kits (Meimian Industrial Co. Ltd., Jiangsu, China) according to the manufacturer’s protocols. To avoid interference of other hormones to the sex hormones, we chose 3-week-old wt (n=12) and tg (n=12) female mice to collect the blood from the orbital sinus vein to detect serum estradiol and progesterone levels. The steps for blood collection are as follows: firstly, female mice were injected with 5 IU pregnant mare serum gonadotropin (PMSG, Sansheng Co. Ltd., Ningbo, China) intraperitoneally, and blood was obtained 48 h after injection to measure serum estradiol level. Simultaneously, female mice were injected with 5 IU human chorionic gonadotropin (hCG, Sansheng Co. Ltd.), and blood was obtained 24 h after injection to measure the serum progesterone level. The blood from the orbital sinus vein of 10-week-old wt (n=12) and tg (n=12) female mice with no further treatment were used to measure the serum prostaglandin E2 level.

### Oocyte collection and evaluation

Cumulus-oocyte complexes (COCs) were collected respectively from wt (n=5) and tg (n=5) female mice. In short, the 3-week-old wt and tg female mice were injected with 5 IU PMSG and 5 IU hCG 48 h later. After 15 h hCG injection, the fallopian tubes were flushed to collect COCs. Then the COCs were washed three times in HEPES-CZB medium and treated with 0.3% hyaluronidase (Sigma, New Jersey, USA) to disperse cumulus layers to collect denuded oocytes. The oocytes were counted, quantified manually and cultured in a CZB medium at 37 °C in a humidified atmosphere of 5% CO_2_-in-air for follow-up detection (Zhu et al., 2015).

### Histological analysis and follicle quantification

The ovaries from 10-week-old wt (n=5) and tg (n=5) mice were weighed, and the relative ratio to mice’s body weight was estimated. The ovaries were fixed in 4% paraformaldehyde overnight, dehydrated by a graded alcohol series, embedded in paraffin, and then serially sectioned (5 µm) using standard procedures. Then, the sections were stained with Haematoxylin and Eosin (HE) solution and examined under a light microscope (BX43, Olympus, Japan) as previously described (Rao et al., 2023a). The ovaries were serially sectioned, and one-fifth to tenth sections were counted for the follicle quantification. To avoid duplicate counting, follicular cells with apparent nuclei were quantified manually, and the follicles were classified as primitive follicles, secondary follicles, early antral follicles, mature follicles, corpus luteum, and atresia follicles (Fransolet et al., 2015). Moreover, the manual surround of the cortex was used to calculate follicular densities (number/mm^2^) by Image J software (NIH, USA) (Fransolet et al., 2015).

### Estrous cycle detection

Ten-week-old wt (n=5) and tg (n=5) female mice were used to detect the estrous cycles for at least 21 consecutive days. The cycle detection method is as described by McLean et al. (2012) and briefly as follows: dropping the vaginal flushing fluid onto the slide, fixing it in cold methanol for 15 minutes, and then drying at room temperature. Then, the slide was stained by using Rui’s Giemsa test kit (Servicebio, Wuhan, China) and coverslip for microscopic visualization on a Nikon microscope. The identification of the mouse estrous cycle (proestrus (P), estrus (E), metestrus (M), and diestrus (D)) was categorized by the presence and relative abundance of leukocytes, nucleated epithelial cells, and cornified squamous epithelial cells on the slide (Cora et al., 2015). All estrous cycle detection was performed at 9:00 a.m. to 10:00 a.m.

### qRT-PCR analysis

Total RNA of ovaries of 3-week-old wt (n=5) and tg (n=5) female mice was extracted using RNAiso Plus reagent (Vazyme, Nanjing, China) and was synthesized to cDNA by reverse transcription PCR (RT-PCR) PrimeSript™ RT reagent Kit (Vazyme). Then, cDNA was used to detect the gene expression level by using qRT-PCR analysis as follows: 10 μL SYBR® Premix Ex Taq II (Vazyme), 1 μL cDNA, 10 μmol/L PCR Forward/Reverse Primer, and added RNase-free water to a total volume of 20 μL. Each sample was run in triplicate. The relative transcriptional level of the target gene was normalized to the 36B4 expression level using the method of 2^−ΔΔCt^ (Livak and Schmittgen, 2001). The specific primers of the Pai gene used in this study were: forward: 5-tgacgagcgggaatacttta-3; reverse: 5-gagggtcatcagcccatcta-3. The specific primers of the 36B4 gene (housekeeping gene) used in this study were: forward: 5-cactggtctaggacccgagaag-3; reverse: 5-ggtgcctctggagattttcg-3.

### Western blot analysis

The 10-week-old ovaries protein of wt and tg mice were lysed by cold RIPA buffer (Beyotime) for 30 min on ice, added to 5× SDS-PAGE loading buffer (Beyotime), and boiled at 100 °C for 10 min. Then, the lysates were separated by 8-12% SDS-PAGE and transferred electrophoretically onto PVDF membrane (Millipore, USA). After being blocked with 5% defatted milk powder in TBS-T buffer (20 mM Tris/HCl pH 8.0, 150 mM NaCl, 0.05% Tween 20), PVDF membranes were incubated with the primary antibodies (His-tag, Proteintech, Wuhan, China). The expression level of the Pai protein was marked by that of His-tag. After incubation with the HRP-conjugated secondary antibody (Santa Cruz, USA), the signals were measured using ECL reagents (Vazyme) using the Chemiluminescent Imaging System (Tanon, China). GAPDH (Proteintech) was used as an endogenous loading control.

### Statistical analysis

All histogram data were analyzed using SPSS 18.0 (SPSS Inc., USA) and were expressed as the means ± SD. ANOVA with Tukey’s HSD post hoc test was applied to multigroup comparisons, whereas the student’s t-test was used for the two-group comparisons. The data were considered statistically significant when the P value was less than 0.05.

## Result

### T10c12-CLA didn’t affect the body and ovary weight of tg female mice

**Figure 1:**
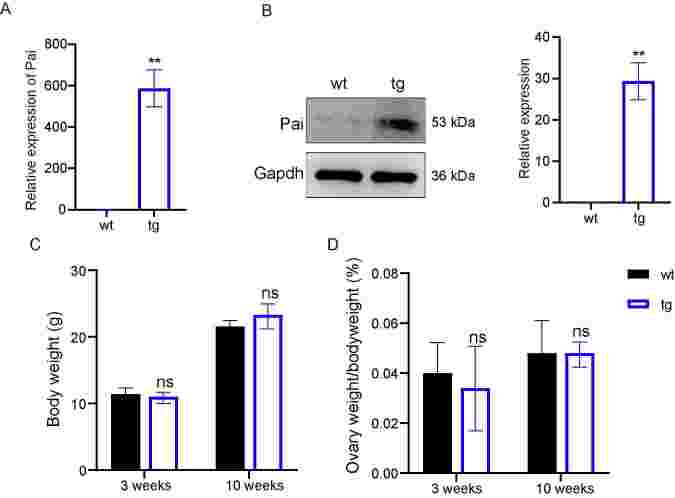
Transgenic (tg) and wild-type (wt) mice had no change in body and ovary weights. (A) qRT-qPCR analysis of Propionibacterium acnes isomerase (*Pai*) gene in ovaries of wt (n=5) and tg (n=5) mice; (B) Western blot analysis of Pai in ovaries of wt and tg mice; (C) The body weight of 3-, and 10-week-old wt and tg mice; (D) The relative ovaries weight to body weight of 3-, and 10-week-old wt and tg mice. ns represents *P*>0.05, and ** represents *P*<0.001.

To test the effect of t10c12-CLA on female reproductive ability, we detected the ovary development using the mice inserting a Pai-expressing cassette into the Rosa26 locus. Firstly, the qRT-PCR analysis displayed that the *Pai* gene was highly expressed in the ovaries of tg mice compared with those of wt mice (Figure 1A). Western blot analysis showed results similar to the qRT-PCR analysis (Figure 1B). These results proved that Pai was successfully inserted and appropriately functioned in the tg ovaries. Moreover, the gas chromatography of adult tg mice demonstrated that the Pai protein successfully converted LA into t10c12-CLA, and fatty acid contents had undergone changes (Rao et al., 2023a). The result predicted that the 10c12-CLA produced by Pai altered the serum fatty acid contents of tg mice. The body and relative ovary weight had no significant difference between tg and wt mice at ages 3 or 10 weeks (Figure 1C, D).

### T10c12-CLA delayed the birth of the first-born of tg female mother

To explore the effect of t10c12-CLA on the reproductive capacity in tg mice, 8-week-old mice were put in the same cage to mate for 7 months according to the methods. The mating results showed that much more offspring were born in the wt mother group than in the tg mother group (Table 1). While there was no significant difference in the litter size in tg (6.05 ± 2.60) and wt (6.77 ± 2.81) mice (Table 1). Statistical analysis of the number of litters revealed that the tg (4.75 ± 2.75) group was less than the wt (6.67 ± 0.57) group (*P*<0.05, Table 1). There was no significant difference in the offspring’ sex ratio per litter in tg ((1.08 ± 0.58):1) and wt ((0.97 ± 0.62):1) mice. Moreover, the latency (in days) to the first litter of the tg mothers (32.0 ± 4.70 d) was significantly prolonged than those of wt mothers (21.3 ± 2.31 d; *P*<0.05, Table 1). Moreover, the two groups did not significantly change the latency (in days) to the other litters. The results showed that the tg mice presented poor reproductive success as the size of the litter in which they were born increased.

**Table 1:** Female fertility in wild-type and transgenic mice.

**Foster mother**	**Wild-type mice**	**Transgenic mice**
Total litters	24	18
Litter size	6.77 ± 2.81	6.05 ± 2.60
The number of litters	6.67 ± 0.57	4.75 ± 2.75
Male to female ratio	(0.97 ± 0.62):1	(1.08 ± 0.58):1
Latency to the first litter (d)	21.3 ± 2.31	32.0 ± 4.70^*^
Latency to the other litter (d)	33.7 ± 14.9	36.8 ± 17.1

* represents *P*<0.05.

### T10c12-CLA reduced the progesterone level and increased oocyte number after superovulation in female mice

**Figure 2:**
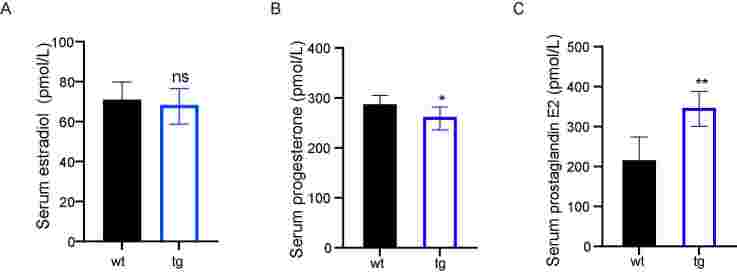
Transgenic (tg) mice exhibited decreased serum progesterone and increased plasma prostaglandin E2 levels than wild-type (wt) mice. The level of serum estradiol (A) and progesterone (B) of 3-week-old wt and tg mice; (C) The level of serum prostaglandin E2 of 10-week-old wt and tg mice. ns represents *P*>0.05, * represents *P*<0.05, and ** represents *P*<0.001.

To explore the effect of t10c12-CLA on the latency (in days) to the first litter on tg mice, we detected the female reproductive hormone secretion, ovarian structure, and estrous cycle. ELISA analysis showed that the serum estradiol level did not change (68.4 ± 8.1 vs. 71.0 ± 8.8 pmol/L; Figure 2A) while the level of serum progesterone was significantly reduced (261.8 ± 19.6vs. 287.1 ± 17.9 pmol/L; *P*<0.05, Figure 2B) in tg mice compared to wt mice. In addition, the level of serum prostaglandin E2 was significantly increased (346.9 ± 40.9 vs. 215.4 ± 58.0 ng/L; *P*<0.05, Figure 2C) in tg mice compared to wt mice. These results suggested that t10c12-CLA might reduce the progesterone level in female mice.

**Figure 3:**
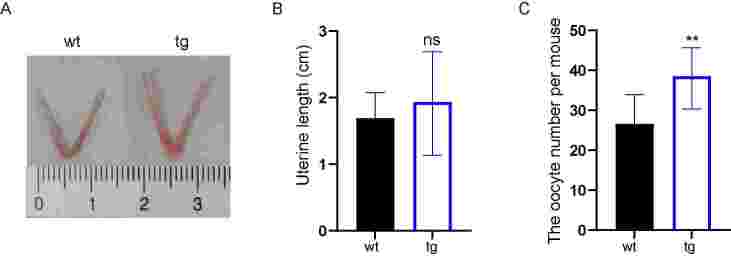
Transgenic (tg) mice showed more oocyte numbers after superovulation than wild-type (wt) mice. (A and B) The uterine length of 3-week-old wt and tg mice; (C) The oocyte number per super-ovulated mouse in 3-week-old wt and tg group. ns represents *P*>0.05, and ** represents *P*<0.001.

The oocyte quality is crucial to embryonic development. We measured the length from the cervix to the bottom of the uterus of 3-week-old mice and found that the uterus length of tg mice slightly increased but was not significant (Figure 3A, B). The oocyte number per super-ovulated mouse was significantly increased in tg mice than that of wt mice (38.6 ± 7.15 vs 26.6 ± 7.32, *P*<0.05, Figure 3C). All the results predicted that t10c12-CLA could influence reproductive potential by down-regulating progesterone secretion and oocyte number in tg female mice.

### T10c12-CLA led to irregular estrus cycles in female mice

**Figure 4:**
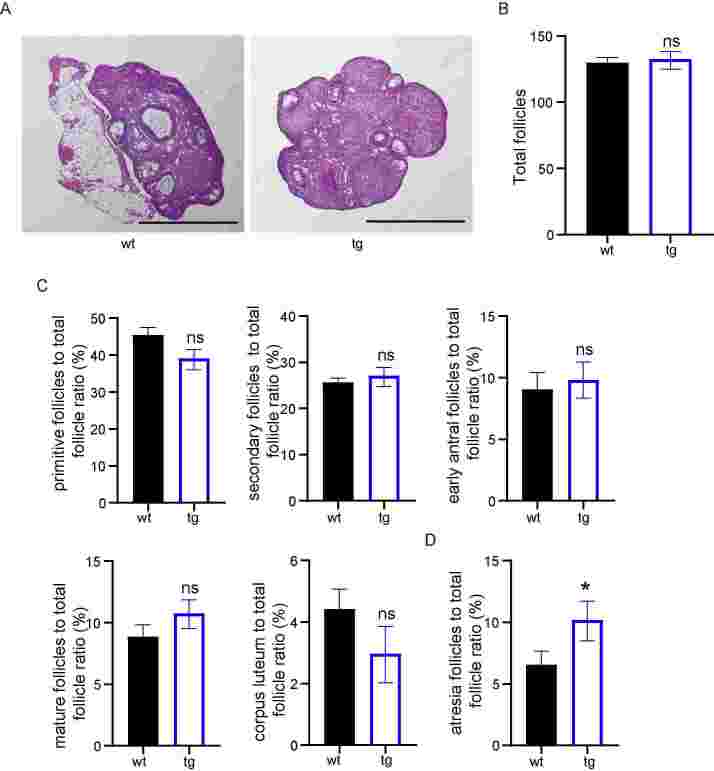
Analyses of follicles from wild-type (wt)and transgenic (tg) ovaries. (A) Haematoxylin and Eosin staining shows ovary structure of wt and tg mice; (B) analyses of the total number of follicles per ovary; (C) the percent of primitive follicles, secondary follicles, early antral follicles, mature follicles, and corpus luteum per ovary from wt and tg mice; (D) the percent of atresia follicles per ovary from wt and tg mice. ns represents *P*>0.05, and * represents *P*<0.05.

We also detected adult mice to observe the long-term effects of t10c12-CLA on the ovaries. Furthermore, ovaries collected from tg and wt mice after 10-week breeding trial exhibited follicles of various sizes in central sections of ovarian tissue in both genotypes (Figure 4A). We observed typical ovarian structures in the tg mice that were well-developed, accompanied by visible follicles developed at various stages, and corpora lutea compared to wt mice. There was no difference in the total number of follicles between wt and tg groups (Figure 4B). In contrast, the numbers of secondary follicles, early antral follicles, and mature follicles in tg mice were slightly increased but not significant (Figure 4C). The atresia follicles proportion of all follicles in tg mice were significantly increased than those in wt mice (Figure 4D). These results predicted that t10c12-CLA could induce follicle atresia in tg mice.

**Figure 5:**
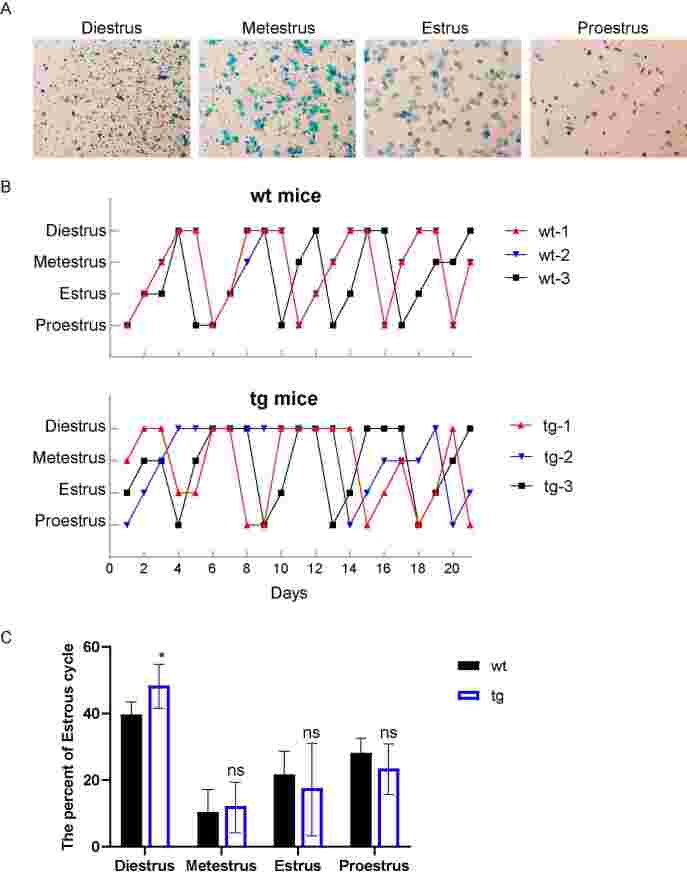
Transgenic (tg) mice predicted estrus cycle disorder compared with wild-type (wt) mice. (A) Smears were stained with crystal violet staining and staged based upon cytology; (B) Estrous cycle stages for representative wt and tg mice analyzed by vaginal smears; (C) The per cent proportion of estrous cycle stage. ns represents *P*>0.05 and * represents *P*<0.05.

The results of reproductive and reproductive hormone testing showed that the tg mice had a delayed initial estrus period and changes in sexual hormone levels. Due to the regulation of hormone levels during the estrous cycle, it is speculated that t10c12-CLA will affect the estrous cycle of mice. Subsequently, 10-week-old female mice were selected to test their estrus cycle. Determine the estrus stage of the mouse based on the shedding of vaginal cells and continuously monitor for 21 days. The estrus cycle is divided into the proestrus, estrus, metestrus, and diestrus stages (Figure 5A). As shown in Figure 5B, the estrus cycle of wt female mice is typically approximately 4~5 days, and the tg mice showed erratic estrous cycle patterns. Then, the estrous cycle analysis was performed in wt and tg females, and the results showed that the diestrus period of tg mice was more extended than those of wt mice (Figure 5C). The results indicated that t10c12-CLA caused an extension of the estrus interval in mice, leading to irregular cycles.

## Discussion

Several studies have been carried out to investigate the effects of CLA on male and female reproductive and metabolic aspects and demonstrated that a mixture of CLA isomers provides some benefit on reproductive parameters (Freitas et al., 2023; Safari Hasanabad et al., 2022). In contrast, it was difficult to determine whether this was the long-term effect of pure t10c12-CLA. In our previous study, we produced a novel tg mouse, which could produce t10c12-CLA itself, solving the difficulty of obtaining pure CLA (Rao et al., 2023a). Then, the female tg mice were used to determine the long-term effect on the female reproductive parameters in the current study. The tg mice showed high prostaglandin E2 level, increased atresia follicles percent, and irregular estrous cycle, leading to the prolonged latency (in days) to the first litter and reduced litter size.

In the current study, t10c12-CLA did not affect relative ovary weight and uterine length, corroborating reports in the literature (Freitas et al., 2022; Knott et al., 2022). When the tg female mice mated with the wt male, we found that the time from mating to the first litter was latency, and the mean offspring number was less in the whole period of 7 months. The result indicated that t10c12-CLA reduced the fertility ability of female mice, which was significantly different from existing studies (Badawy et al., 2023; Freitas et al., 2022). The result might be due to the higher purity of t10c12-CLA than the CLA mixture during feeding. Meanwhile, t10c12-CLA could be directly synthesized and secreted in the ovaries and placenta, so that could be directly participating in related biological processes. However, the amount of CLA entering the ovaries and placenta during feeding was unknown. Furthermore, our findings focus much more on the impact of long-term t10c12-CLA existence on individuals. These fertility results were closely related to changes in sex hormones.

Simultaneously, we detected the changes in serum hormones and found higher prostaglandin E2 levels and lower progesterone levels in tg mice. T10c12-CLA could change the concentration of arachidonic acid in the blood, which was the precursor for synthesizing prostaglandin E2 (Hashimoto et al., 2022). That is, the level of serum prostaglandin E2 could be easily upregulated by t10c12-CLA.

The secretion of hormones determines the physiological characteristics of the body. Prostaglandin E2 orchestrated ovulation processes, including cumulus expansion, oocyte release, follicle rupture, and angiogenesis, via its four receptor types present in the ovary (Kim and Duffy, 2016; Lundberg et al., 2020). Moreover, Prostaglandin E2 also induced ovulation and spawning in unfertilized individuals, which had a bilayer follicular structure compared to monolayer follicular in the perinatal period black rockfish (Lyu et al., 2024). Progesterone allowed the endometrial transition from a proliferative to the secretory stage, facilitated blastocyst nesting, and was essential to the maintenance of pregnancy (Taraborrelli, 2015). Then, we present a global analysis of the female reproductive system through ovarian structure, oocyte discharge, and estrus cycle.

The oocyte number per super-ovulated mouse was significantly increased in tg mice than that of wt mice, similar to the effect of t10c12-CLA in black rockfish (Lyu et al., 2024). The increase in oocyte number predicted a reduction in female donors and higher donor utilization (Luo et al., 2011). Of course, whether a large number of super-ovulated oocytes also indicated an advantage in quality requires further exploration. The result reflected the other potential role of t10c12-CLA in addition to fat metabolism.

Moreover, serum hormonal disorder ultimately led to an irregular estrous cycle. The normal estrous cycle of mice was 2-7 days (Kim et al., 2023). If a mouse spends more than 50% of its estrous cycle during a particular estrous stage, or if its average estrous cycle length was 7 days or more, it was considered to have a disordered estrous cycle. The disordered estrus cycle led to prolonged pregnancy time of the first mouse fetus. With the continuous reproduction experiment, wt female mice demonstrated sustained and stable reproductive ability. Still, tg mice had a decrease in the number of offspring starting from the third litter, and during the process, 50% of the mother mice stopped reproducing. All female mice had significantly longer days of first birth. Therefore, it was inferred that tg female mice had reproductive ability and could generally have offspring and breastfeed but did not have an advantage in continuous reproduction experiments. It was speculated that this change in serum fatty acid composition will affect the steroid hormone synthesis and ultimately affect female fertility by affecting the cholesterol content. The results suggested that the chronic effects of t10c12-CLA on female mice, to a certain extent, would impact female fertility.

## Conclusion

Taken together, t10c12-CLA could affect progesterone biosynthesis, cause estrous cycle disorder and severe follicular atresia, and inhibit the reproductive performance of female mice.
